# Investigating Treatment Response in Obsessive–Compulsive Disorder Through Neuromodulation and Patient-Derived Induced Pluripotent Stem Cell Models: Preliminary Clinical Observations from a Translational Study

**DOI:** 10.3390/brainsci16050537

**Published:** 2026-05-19

**Authors:** Beatrice Benatti, Matteo Marcatili, Rodolfo Leuzzi, Massimo Clerici, Luciano Conti, Massimo Gennarelli, Carlo Sala, Federico Bernoni d’Aversa, Valentina Casati, Michele Castiglioni, Camilla Gesi, Nicolaja Girone, Luca Larini, Alessandra Minelli, Emma Rinaldi, Matteo Vismara, Bernardo Dell’Osso

**Affiliations:** 1 Department of Health Sciences, Università degli Studi di Milano, 20122 Milan, Italy; beatrice.benatti@unimi.it; 2 CRC ‘Aldo Ravelli’ for Neurotechnology & Experimental Brain Therapeutics, 20122 Milan, Italy; 3 Department of Biomedical and Clinical Sciences, Psychiatry Unit 2, ASST Fatebenefratelli-Sacco, Università degli Studi di Milano, 20157 Milan, Italy; rodolfo.leuzzi@unimi.it (R.L.);; 4 Department of Mental Health, Fondazione IRCSS San Gerardo dei Tintori, 20900 Monza, Italy; matteo.marcatili@irccs-sangerardo.it; 5 Department of Medicine and Surgery, Università degli Studi di Milano-Bicocca, 20126 Milan, Italy; massimo.clerici@unimib.it; 6 Department of Cellular, Computational and Integrative Biology—CIBIO, University of Trento, 38122 Trento, Italy; luciano.conti@unitn.it; 7 Dipartimento di Medicina Molecolare e Traslazionale, Università di Brescia, 25121 Brescia, Italy; 8 Unità di Genetica, IRCCS Istituto Centro San Giovanni di Dio Fatebenefratelli, 25125 Brescia, Italy; 9 CNR Istituto di Neuroscienze, 20854 Vedano al Lambro, Italy; 10 Department of Psychiatry and Behavioural Sciences, Bipolar Disorders Clinic, Stanford University, Stanford, CA 94305, USA

**Keywords:** obsessive–compulsive disorder, treatment-resistant OCD, neuromodulation, transcranial magnetic stimulation, induced pluripotent stem cells, medium spiny neurons, translational psychiatry

## Abstract

**Highlights:**

**What are the main findings?**

- A translational framework integrating clinical stratification and patient-derived hiPSC neuronal models in OCD is presented.
- Preliminary clinical observations indicate early symptom improvement following theta-burst stimulation (TBS) in a subset of treatment-resistant OCD patients.

**What are the implications of the main findings?**

- Patient-derived neuronal models may enable the exploration of molecular patterns associated with differential treatment response in OCD.
- This approach provides a framework for investigating biological mechanisms underlying treatment resistance in OCD.

**Abstract:**

Background: Treatment-resistant obsessive–compulsive disorder (OCD) is a heterogeneous and clinically challenging condition. Growing evidence suggests alterations in glutamatergic signaling within cortico–striatal–thalamo–cortical circuits, including those involving medium spiny neurons (MSNs), as well as genetic factors affecting synaptic organization, although the biological mechanisms underlying differential treatment response remain incompletely understood. Methods: This multicenter study presents a translational research framework aimed at investigating potential molecular and cellular correlates of treatment response in a cohort of patients with OCD, stratified according to their response to pharmacological treatments and transcranial magnetic stimulation (TMS). Peripheral blood mononuclear cells from clinically defined subgroups are reprogrammed into human induced pluripotent stem cells and differentiated into MSN-enriched neuronal cultures, enabling in vitro investigation of morphological, biochemical, and transcriptomic features associated with different clinical profiles. Optogenetic and pharmacological stimulation paradigms are applied to probe selected aspects of neuronal activation in vitro, providing a controlled and simplified experimental framework to explore cellular responses under different treatment conditions. By integrating clinical phenotyping with patient-derived cellular models, this study establishes a translational platform for hypothesis generation in the investigation of treatment response in OCD. Results and Conclusions: Preliminary clinical observations from an initial cohort undergoing neuromodulation are also reported to document feasibility and early clinical implementation of the study, providing an initial characterization of the cohort.

## 1. Background

Obsessive–compulsive disorder (OCD) is a chronic, disabling psychiatric disorder, characterized by obsessions and compulsions, causing significant impairment and clinical distress in patients [1]. This condition may have a substantial impact on quality of life (QoL), affecting multiple domains of functioning, including work, social, and family environments [2].

The etiology of OCD has not been fully elucidated; however, growing evidence suggests alterations in glutamatergic signaling in the cortico–striatal–thalamo–cortical (CSTC) circuitry, including those involving medium spiny neuron (MSN) activity, although the causal mechanisms remain to be established [3,4]. The typical conceptualization of this model involves a direct and indirect pathway: the direct, excitatory pathway is modulated by the indirect pathway’s inhibitory function. In OCD, a lower threshold of activation of this system has been hypothesized to contribute to increased activity in the direct pathway compared to the indirect pathway, within a broader context of CSTC circuit dysregulation, leading to a hyperactivation of the orbitofrontal-subcortical pathway [5]. In CSTC circuitry, the striatum modulates the pathway through the coordinated activity of two classes of GABAergic inhibitory MSNs, which represent 95% of the cells in the striatum. There are two main phenotypes of MSNs: in the direct pathway, MSNs expressing D1 receptors are predominant, whereas the indirect pathway consists mainly of MSNs expressing D2 receptors. Because the activity of D1- and D2-MSNs depends on the integration of cortical glutamatergic inputs and dopaminergic modulation, among other neuromodulatory influences, alterations in glutamate transmission can shift the balance between the direct and indirect pathways. Such imbalance may contribute to the hyperactivity of the CSTC circuitry and, consequently, to the manifestation of OCD symptomatology [6].

Despite evidence for substantial heritability, robust and reproducible gene-level associations have only begun to emerge in recent large GWAS and multi-omics efforts [7]. While convergence remains modest for individual genes, it is suggestive at the level of biological pathways rather than individual genes, with recurrent signals implicating synaptic organization, plasticity and transmission within CSTC-relevant circuits [8,9,10]. Among these, the SAPAP family of postsynaptic scaffold proteins—encoded by the DLGAP1–4 gene cluster—has emerged as particularly relevant in both human and animal research. SAPAP proteins play a key role in regulating glutamate receptor trafficking and their localization at the postsynaptic density. In particular, SAPAP3, encoded by DLGAP3, is especially abundant in the striatum, and the loss of SAPAP3 disrupts the NMDA–SHANK–SAPAP complex, impairing postsynaptic glutamatergic signalling and scaffold stability [11]. SAPAP3 knockout mice show compulsive grooming, increased anxiety-like behaviors, and cortico–striatal synaptic abnormalities, mirroring core features of OCD [12,13].

Although the underlying mechanisms are not fully understood, disruptions in AMPA receptor–mediated transmission in striatal medium spiny neurons have been proposed as a contributing factor.

More recent evidence has also highlighted the SLITRK family of transmembrane proteins, which regulate synapse formation and maintenance. SLITRK5, in particular, is highly expressed in striatal neurons, and SLITRK5 knockout mice exhibit selective cortico–striatal alterations accompanied by compulsive-like behaviors. Furthermore, recent large exome case–control analyses have identified SLITRK5 as one of the most prominent gene-level signals, although approaching, but not reaching, the exome-wide significance threshold for an excess burden of rare damaging variants [14].

Together, these findings suggest that genetic variants affecting synaptic organization—such as those involving DLGAP3 (SAPAP3) and SLITRK5—may contribute to vulnerability to OCD, although these associations remain to be fully established [13,15].

Pharmacological treatment of obsessive–compulsive disorder primarily targets monoaminergic neurotransmission, with a predominant focus on the serotonergic system. Serotonin reuptake inhibitors (SRIs) represent the first-line pharmacological treatment and are typically administered at higher doses than those used in other psychiatric conditions, with subsequent titration according to clinical response and tolerability. Following sustained remission, the continuation of pharmacotherapy may be reassessed jointly by clinicians and patients [16,17]. Despite these strategies, approximately 40–60% of patients are estimated to fail to achieve an adequate response, meeting criteria for treatment-resistant OCD (TR-OCD). In these cases, neuromodulation approaches targeting CSTC circuits, such as transcranial magnetic stimulation (TMS), have gained increasing clinical relevance [18]. These approaches include theta burst stimulation (TBS), a high-frequency variant of TMS that can be applied to cortical regions implicated in OCD pathophysiology and deep TMS (d-TMS), which targets medial prefrontal and anterior cingulate regions [19,20,21,22,23].

Advances in cellular reprogramming techniques now allow the generation of human induced pluripotent stem cells (hiPSCs) from patient-derived somatic cells, which can be differentiated into specific neuronal subtypes, including striatal MSNs [24,25]. This approach enables the development of patient-specific cellular models that may be used to explore molecular and cellular features associated with neuropsychiatric conditions.

In this context, integrating detailed clinical phenotyping with hiPSC-derived neuronal models may provide a translational framework to explore molecular correlates of differential treatment response in OCD. Accordingly, the present multicenter study is designed to investigate treatment-response profiles in OCD by combining clinical stratification, neuromodulation, and patient-derived cellular modeling within an exploratory framework.

## 2. Methods

### 2.1. Specific Aims and Study Design

The study was designed in accordance with reporting recommendations (SPIRIT 2013 Statement) to ensure transparency and methodological rigor [26] and is designed as a multicenter translational investigation integrating clinical stratification with patient-derived cellular models, supported by preliminary clinical observations.

The main objectives of the study are:

(i) To characterize patients with OCD stratified according to their clinical response or non-response to pharmacological treatment with serotonin reuptake inhibitors (SRIs).
(ii) To apply two neuromodulation protocols—Theta-Burst Stimulation (TBS) and Deep Transcranial Magnetic Stimulation (d-TMS)—with the aim of stratifying patients based on their clinical response.
(iii) To generate patient-derived human induced pluripotent stem cells (hiPSCs) and differentiate them into neuronal populations enriched in striatal medium spiny neurons (MSNs), in order to establish an in vitro platform to explore morphological, biochemical, and transcriptomic features potentially associated with different treatment-response profiles.

Patients are recruited at the Obsessive–Compulsive Disorder Outpatient Clinic of Psychiatry Unit 2, Luigi Sacco Hospital (ASST Fatebenefratelli Sacco, Milan, Italy), and at the Fondazione IRCCS San Gerardo dei Tintori, Monza. Following a complete description of the study procedures, all participants will provide written informed consent. At enrollment, eligibility will be confirmed through the administration of the Structured Clinical Interview for DSM-5 (SCID-5). For each participant, clinical and sociodemographic information will be systematically collected using a dedicated anonymized electronic case report form (eCRF). Recorded variables will include sex, age, education, occupation, social status, dominant hand, body mass index, medical comorbidities, and family history. Additional clinical data—such as age at onset, illness duration, duration of untreated illness, and changes in pharmacological treatment—will also be documented. The assessment phase will be completed through the administration of standardized psychometric scales.

### 2.2. Sample Size and Eligibility Criteria

A total of 60 patients diagnosed with OCD according to DSM-5 TR criteria will be recruited at the two aforementioned psychiatric units. The initial objective of the study is to classify participants according to their pharmacological response. Accordingly, participants will be stratified into two groups:

- 20 OCD patients who show a clinical response to serotonin reuptake inhibitors (SRI) treatment—responders [R];
- 40 OCD patients who do not respond to SRI—treatment-resistant [TR].

Given the exploratory and translational nature of the study, this sample size was selected to allow clinical stratification and feasibility of subsequent cellular analyses, rather than to provide adequately powered comparisons between groups.

The inclusion criteria will be the following: diagnosis of Obsessive–Compulsive Disorder (according to DSM-5-TR [1]); both sexes; age ≥ 18 years and ≤65 years, ability to provide valid written informed consent; for patients classified as responders to pharmacological treatment, a clinical history of at least one pharmacological trial with an SRI for a minimum of 6 weeks and evidence of treatment response, defined as a ≥30% reduction in the Yale–Brown Obsessive Compulsive Scale (Y-BOCS) score [27] relative to the patient’s baseline; for patients classified as resistant to pharmacological treatment [TR], clear evidence of treatment resistance, defined as the absence of a significant clinical response after at least two treatment trials with selective serotonin reuptake inhibitors (SSRIs) and one trial with clomipramine, each administered for a minimum of 12 weeks at the maximum recommended dose [28].

Exclusion criteria will include inability to provide informed consent; no clinical history of treatment with SRI medications; clinical history of epilepsy or seizures; presence of a pacemaker, removable metal prostheses, implanted medical pumps, or intracranial metal clips.

Both inclusion and exclusion criteria are reported in Table 1.

### 2.3. Neuropsychological and Psychiatric Assessment

Participants will undergo a comprehensive clinical and neuropsychological assessment through the administration of standardized psychometric instruments: Yale–Brown Obsessive Compulsive Scale (Y-BOCS, [27]), Hamilton Depression Rating Scale (HDRS-21, [29]), Hamilton Anxiety Rating Scale (HARS, [30]), Mini-Mental State Examination (MMSE; [31]), Cambridge Neuropsychological Test Automated Battery (CANTAB; [32]), and Clinical Global Impression (CGI; [33]).

### 2.4. Treatment Administration

The second objective of the study is to apply and evaluate two neuromodulation protocols—deep Transcranial Magnetic Stimulation (d-TMS) and Theta-Burst Stimulation (TBS)—and to classify participants according to their clinical response. Both protocols follow established safety guidelines [34].

Forty patients in the TR-OCD group will be randomized in a 1:1 ratio to receive either TBS or d-TMS, using a stratified randomization scheme generated through SPSS version 23 (Statistical Package for the Social Sciences). Randomization is adopted to minimize allocation bias and to ensure balanced distribution of patients across neuromodulation protocols. The study is not designed or powered to compare the clinical efficacy of the two stimulation techniques but rather to enable stratification based on early treatment response for subsequent cellular analyses. For each participant, Y-BOCS scores (baseline and follow-up) and clinical response characteristics will be collected. Clinical response will be defined as a reduction of at least 20% in the total Y-BOCS score at T1 compared to baseline. Partial response will be defined as a Y-BOCS reduction between 20% and 30% and full response as a Y-BOCS reduction > 30%. These thresholds are selected to capture early clinical changes within a short observational window and are not intended to reflect standard definitions of treatment response, but rather to support patient stratification for downstream analyses. These thresholds are used exclusively for stratification purposes within the study design and should not be interpreted as indicators of clinically meaningful response at this stage.

All patients will maintain a stable pharmacological regimen throughout the neuromodulation phase.

#### 2.4.1. d-TMS Protocol

The d-TMS protocol will be conducted at IRCCS San Gerardo Monza and will consist of 5 weeks of daily sessions (5 days per week), followed by 4 sessions during the 6th week and a 4-week follow-up period, in line with recent clinical studies. Stimulation will be delivered using a TMS device equipped with an H-coil [35]. The coil will be positioned 4 cm anterior to the foot motor cortex and set at 100% of the leg resting motor threshold (RMT), defined as the minimal intensity eliciting involuntary foot contractions in 3 out of 6 attempts. This placement targets the dorsal medial prefrontal cortex (mPFC) and anterior cingulate cortex (ACC) bilaterally. Each session will consist of 20 Hz stimulation at 100% RMT, delivered in 2 s trains with 20 s intertrain intervals, for a total of 50 trains and 2000 pulses. Non-response will be defined as a reduction in less than 20% in Y-BOCS scores relative to baseline.

#### 2.4.2. TBS Protocol

The TBS protocol will be conducted at the OCD Clinic, ASST Fatebenefratelli-Sacco, and will consist of 1 week of daily sessions followed by a 4-week follow-up, allowing the evaluation of early clinical changes after treatment initiation. Stimulation will be delivered using a Magstim Rapid2 device. Continuous TBS will be applied to the left orbitofrontal cortex using 3-pulse 50 Hz bursts repeated every 200 ms (5 Hz) at 80% of the active motor threshold, defined as the minimal intensity required to elicit a right thumb movement during stimulation of the left primary motor cortex. The stimulation site will be identified using the Fp1 location of the international 10–20 EEG positioning system as a scalp-based reference for the left orbitofrontal region. This scalp-based positioning approach has been used in previous orbitofrontal TBS studies and allows a practical and reproducible approximation of the target region in clinical settings where neuronavigation is not routinely available [20], although it may introduce variability in cortical target localization across individuals and should be considered a methodological limitation. Treatment will consist of two sessions per day, 30 min apart, for 5 consecutive days. Each session will deliver 600 pulses over 40 s [36], for a total of 1200 pulses per day. Non-response will be defined as a reduction in less than 20% in Y-BOCS scores at follow-up compared with baseline.

#### 2.4.3. Patients Stratification

Following treatment with d-TMS or TBS, participants will be further stratified into four subgroups based on their clinical response:

1. d-TMS R: SRI treatment-resistant OCD patients who show a clinical response to d-TMS;
2. TBS R: SRI treatment-resistant OCD patients who show a clinical response to TBS;
3. d-TMS NR: SRI treatment-resistant OCD patients who do not show a clinical response to d-TMS;
4. TBS NR: SRI treatment-resistant OCD patients who do not show a clinical response to TBS.

A fifth group will consist of OCD patients who show a clinical response to SRI treatment (SRI responders, [R]), identified during the initial stratification phase.

### 2.5. Generation of hiPSCs and Molecular Analyses

The third aim of the study is to explore potential alterations in MSNs that may be associated with OCD and differential treatment response. To this end, three clinically defined groups will be compared: (1) SRI treatment-responders (SRI-R); (2) SRI-resistant patients who show a clinical response to TMS (SRI-TR-TMS-R); and (3) SRI-resistant patients who do not show a clinical response to TMS (SRI-TR-TMS-TR). All groups will be compared with age-matched healthy controls.

Given the exploratory and translational nature of this part of the study, the cellular analyses are intended to identify preliminary signals rather than to establish definitive mechanistic conclusions.

A peripheral venous blood sample will be collected from three participants in each group using BD Vacutainer CPT tubes, which allow the isolation of peripheral blood mononuclear cells (PBMCs). Samples will be processed at the Genetics Unit of the IRCCS Istituto Centro San Giovanni di Dio Fatebenefratelli (Brescia), where PBMCs will be isolated by Ficoll density-gradient centrifugation, cryopreserved in liquid nitrogen, and stored for subsequent analyses.

#### 2.5.1. Production of hiPSCs

PBMCs isolated from clinically defined groups of patients (SRI-R; SRI-TR-TMS-R; SRI-TR-TMS-TR) will be reprogrammed into footprint-free hiPSC lines using a combination of the four reprogramming factors (OCT4, c-MYC, KLF4, SOX2) through a non-integrating Sendai virus–based vector system (Life Technologies, Waltham, MA, USA) [37]. This approach enables efficient reprogramming while avoiding integration of the viral genome into the host cell genome [38].

Only hiPSC clones meeting established quality control criteria (e.g., pluripotency markers, genomic integrity, and absence of residual viral elements) will be selected for subsequent procedures.

#### 2.5.2. Generation and Characterization of MSNs

Selected hiPSC clones (3 from each OCD subject) will be differentiated into neural progenitor cells (NPCs) and subsequently into neuronal populations enriched in striatal medium spiny neurons. Mature neuronal cultures will be characterized at both morphological and molecular levels, including the evaluation of neurite and dendritic architecture, as well as the expression of markers associated with neuronal subtypes and synaptic proteins.

Differentiation protocols based on established methods [39] will be used to generate lateral ganglionic eminence progenitor-like cells (LGEPs), which can subsequently be matured into MSN-enriched neuronal populations. While these protocols allow the generation of relatively large neuronal batches, yields and cellular composition may vary across lines and experiments.

Marker expression (e.g., FOXG1, GSX2, CTIP2, FOXP1, FOXP2) will be assessed to confirm lineage specification, while acknowledging that the resulting cultures represent enriched neuronal populations and may retain a degree of heterogeneity.

Overall, these in vitro models will be used to explore potential cellular and molecular features associated with different clinical profiles, within an exploratory and hypothesis-generating framework.

#### 2.5.3. Morphological and Biochemical Changes Induced by Optogenetic and Pharmacological Stimulations of MSNs

To explore cellular responses to controlled neuronal activation, we will examine morphological and biochemical changes induced by optogenetic stimulation in MSNs derived from OCD patients.

Optogenetic approaches have been widely used to investigate activity-dependent neuronal processes in experimental systems, including models of compulsive behaviors [40]. Neural activation will be achieved using an adeno-associated virus serotype 9 (AAV9) carrying the Channelrhodopsin-2 (ChR2) gene under the neuronal Synapsin promoter [41,42].

In this study, optogenetic stimulation provides a controlled in vitro framework to model cell-autonomous aspects of neuronal activation and to investigate intracellular signaling and activity-dependent molecular responses. Within the broader translational design of the study, this approach is used to probe cellular responses to controlled stimulation paradigms that may be relevant to mechanisms underlying neuromodulation-based treatments.

By applying standardized stimulation paradigms, this system enables the comparison of molecular responses across patient-derived neuronal lines, with the aim of identifying patterns potentially associated with differential clinical responses.

To complement optogenetic stimulation, MSN cultures will also be exposed to pharmacological modulation using dopaminergic agonists (e.g., SKF 38393, quinpirole) and antagonists (e.g., haloperidol and clozapine which has a broad receptor profile [43]), in order to explore differential cellular responses under controlled conditions [44].

Morphological and biochemical responses will be assessed by evaluating phosphorylated CREB levels, synaptic marker expression, and neuronal protein changes, including through Western blot analysis [45,46,47]. These approaches may also be influenced by factors such as transduction efficiency and neuronal maturation.

#### 2.5.4. Transcriptomic Profiling and Bioinformatic Analysis

The transcriptome comprises the full set of RNA molecules expressed within a given cellular population and plays a central role in regulating cellular identity, structure, and function, including both coding and non-coding RNAs [48].

Transcriptomic and bioinformatic analyses will be used to explore RNA expression patterns and pathways potentially associated with different treatment conditions in patient-derived neuronal models. Our group has previously investigated transcriptional profiles in relation to antidepressant treatment [49].

Using RNA sequencing (RNA-seq), we will obtain a broad molecular characterization of MSNs derived from the clinical groups described above and from age-matched controls [50]. Transcriptomic profiles will be evaluated under baseline conditions and following optogenetic stimulation, which is used here as a controlled experimental paradigm to induce neuronal activation in vitro.

Transcriptional profiles of MSNs derived from SRI responders and treatment-resistant patients (5–9 MSN colonies per group) will be compared with those of controls to identify candidate differentially expressed genes (DEGs). Pathway analyses will be performed using resources such as PharmGKB and Reactome, with the aim of exploring biological pathways that may be relevant to treatment response variability [51].

Exploratory longitudinal analyses will also be conducted by comparing transcriptomic profiles before and after optogenetic stimulation in TMS-responsive (SRI-TR-TMS-R) and TMS non-responsive (SRI-TR-TMS-TR) groups. These analyses are intended to identify potential molecular patterns associated with differential cellular responses under controlled conditions.

Gene Ontology (GO) enrichment analyses will be performed to explore overrepresented cellular components and biological processes. Enrichment will be evaluated using standard GO annotations, with adjusted *p*-values used as a descriptive criterion for ranking results rather than for confirmatory inference. To further characterize synapse-related features, SynGO analysis will be performed, leveraging a curated ontology focused on synaptic biology [52].

Selected DEGs and pathway-related genes will be further examined using qRT-PCR as an orthogonal validation approach, although not accounting for inter-individual variability.

Overall, this strategy is intended to provide an exploratory molecular overview of transcriptional patterns in patient-derived neuronal models across different clinical groups. At the current stage, these analytical approaches should be considered as planned and exploratory, and their implementation will depend on data availability and sample size.

### 2.6. Statistical Analysis

Statistical analyses will be performed using GraphPad Prism (version 8) and R-based packages where appropriate.

Given the interim and exploratory nature of the current dataset, as well as the limited number of biological samples, analyses will be primarily descriptive and hypothesis-generating rather than confirmatory. Sample sizes for biochemical, morphological, imaging, and transcriptomic experiments are based on feasibility considerations and previous experience in similar experimental settings, typically including multiple independent culture preparations for each condition.

Descriptive statistics will be used to summarize observed patterns across groups. Where appropriate, statistical tests may be applied; however, results will be interpreted with caution in light of the exploratory design and sample size and may be influenced by variability across patient-derived lines.

RNA sequencing data will be analyzed using standard pipelines (e.g., DESeq2), and differential expression analyses will be used to identify candidate genes and pathways of potential interest. Adjusted *p*-values will be reported to rank findings within an exploratory framework.

Pathway enrichment analyses will be performed using established tools (e.g., clusterProfiler), with the aim of exploring biological processes potentially associated with observed transcriptional patterns.

As the study progresses and the full cohort becomes available, more robust inferential analyses will be performed to formally test emerging hypotheses.

Overall, all analyses will be interpreted within an exploratory framework at the current stage of the study.

### 2.7. Ethical Approval and Participant Consent

The study was approved by the Research Ethics Committee of Milan, Area 1 (approval number 0008265/2023, approved on 8 February 2023).

The study is conducted in accordance with the ethical standards of the relevant national and institutional committees on human experimentation and with the principles of the Declaration of Helsinki [53].

All participants provide written informed consent prior to participation in the study.

### 2.8. Organizational Framework and Study Workflow

The study is planned over a total duration of three years. Patient enrollment, treatment administration, and clinical follow-up procedures will be conducted during the initial phase of the project, while experimental activities involving patient-derived cellular models will be carried out in parallel with clinical procedures and throughout the duration of the study.

The study is based on a multicenter collaboration integrating clinical and basic research expertise. Two clinical sites—ASST Fatebenefratelli Sacco (Luigi Sacco Hospital, Milan, Italy) and Fondazione IRCCS San Gerardo dei Tintori (Monza, Italy)—are responsible for patient recruitment, clinical assessment, pharmacological management, and neuromodulation procedures. A dedicated basic research center—the IRCCS Istituto Centro San Giovanni di Dio Fatebenefratelli (Brescia, Italy)—is responsible for the generation of human induced pluripotent stem cells and their differentiation into neuronal populations, as well as downstream molecular and cellular analyses.

This organizational framework enables close integration between clinical phenotyping and experimental investigation, supporting the translational scope of the study.

## 3. Preliminary Clinical Observations

In this interim analysis, preliminary clinical observations from an initial cohort of patients treated with cTBS are reported. These observations are included to document feasibility and early clinical implementation of the study. Patients were recruited in accordance with the inclusion and exclusion criteria described above. Since not all participants had completed the T2 follow-up evaluation at the time of this interim analysis, results are reported for T0 (baseline; first day of treatment) and T1 (one week after treatment initiation).

Clinical response was operationally defined as a reduction of at least 20% in the total Y-BOCS score at T1 compared to baseline. Partial response was defined as a Y-BOCS reduction between 20% and 30%, and full response as a Y-BOCS reduction > 30%, consistent with the stratification approach described above.

To date, 19 patients with treatment-resistant OCD have been enrolled and have undergone the cTBS protocol. Two participants discontinued the study prior to completing treatment (one due to headache and one due to flu-like symptoms) and were excluded from this preliminary analysis. Accordingly, data from 17 participants were included.

Mild and transient headaches were reported during treatment, while no severe adverse events were observed.

A reduction in Y-BOCS and HAM-A scores was observed at T1 compared to baseline (Y-BOCS: 23.1 ± 6.8 at T0 vs. 20.6 ± 7.7 at T1; HAM-A: 12.5 ± 7.6 at T0 vs. 10.9 ± 7.3 at T1).

**Figure 1:**
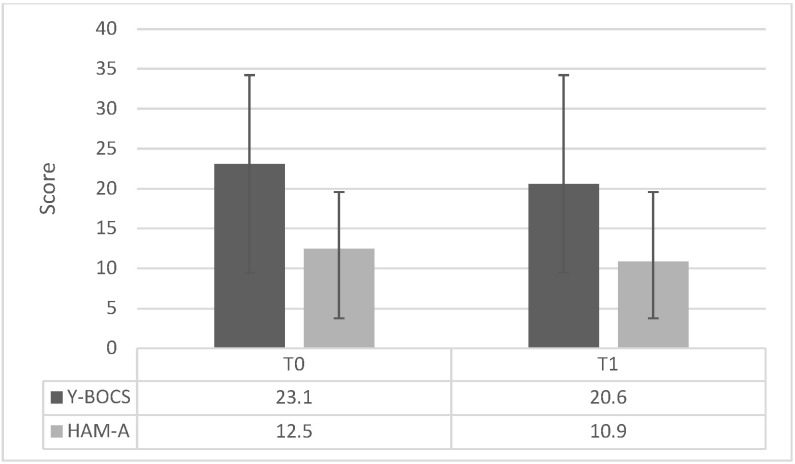
Mean Y-BOCS and HAM-A scores at baseline (T0) and after one week of treatment (T1) in the preliminary cohort (*n* = 17). Values are reported as mean scores.

Based on the predefined criteria, partial responders and full responders represented 23.5% (*n* = 4) and 11.8% (*n* = 2) of the sample (*n* = 17), respectively (see Table 2 and Figure 1).

## 4. Discussion and Conclusions

The present work integrates a structured translational design with preliminary clinical observations. At the current stage, the study provides a structured translational framework accompanied by preliminary clinical observations, while molecular and cellular results are still being generated.

Preliminary clinical observations from the ongoing recruitment phase indicate the feasibility of implementing the neuromodulation protocol and the clinical stratification approach. In a preliminary cohort of treatment-resistant OCD patients undergoing cTBS, a reduction in Y-BOCS scores was observed after one week of treatment, with approximately one third (6 out of 17) of participants meeting criteria for early clinical response. These observations are based on a limited sample and short follow-up period and should be interpreted as descriptive findings, without inferential implications. Clinical response to neuromodulation in OCD is highly heterogeneous, and reliable biological predictors of treatment response remain poorly characterized. In this context, patient-derived neuronal models offer a promising approach to explore molecular patterns potentially associated with differential responsiveness to neuromodulation. In particular, controlled in vitro stimulation paradigms, such as optogenetic approaches, may allow the investigation of activity-dependent cellular responses relevant to neuromodulation within a simplified experimental framework.

The present multicenter study integrates clinical stratification of OCD treatment-response profiles with patient-specific cellular modeling using hiPSCs. By combining SRI responders, TMS-stratified treatment-resistant patients, and molecular analyses of MSN-enriched neuronal cultures, the study addresses a relevant gap in current OCD research, namely the limited understanding of biological processes associated with treatment resistance.

To our knowledge, this is among the first efforts to apply hiPSC-based modeling to OCD within both a psychopharmacological and neuromodulation framework. This approach enables the investigation of molecular, phenotypic, and functional features in patient-derived neuronal systems, providing a platform to explore how cellular processes may relate to variability in clinical response.

The stratification of patients based on treatment response represents a key methodological feature of the study, as it may facilitate the identification of molecular patterns associated with differential responsiveness. However, several limitations should be acknowledged, including the relatively small sample size for cellular analyses, which may limit generalizability and the identification of consistent differences across patient-derived lines, the interim nature of the clinical dataset, and the challenges inherent in translating in vitro findings to clinical phenotypes. At the current stage, clinical, cellular, and molecular components should be considered as parallel and complementary rather than fully integrated. The small sample size of the interim cohort (*n* = 17) substantially limits statistical power, particularly for subgroup analyses, and may introduce bias due to early dropouts. In this context, observed short-term changes in clinical scores should be interpreted cautiously, as they may reflect non-specific effects, including placebo responses and natural clinical variability. The absence of a sham-controlled condition and the limited follow-up period further constrain the interpretability and generalizability of these findings.

Similarly, in the cellular component, the limited number of independent donors (*n* = 3 per group) may increase susceptibility to line-specific variability and technical effects, which cannot be fully disentangled at this stage. Transcriptomic analyses are therefore considered exploratory, and no predefined pathways were used for confirmatory testing to reduce the risk of overinterpretation.

These limitations reflect the early, feasibility-oriented stage of the study. As recruitment progresses within the multicenter framework, increased sample size and extended follow-up will allow more robust analyses and more reliable integration across clinical and molecular levels.

Within these constraints, the study provides a structured framework for generating hypotheses that may inform future mechanistic and translational research.

Overall, this study aims to integrate clinical and cellular dimensions of OCD, contributing to a more refined understanding of treatment response.

## Figures and Tables

**Table 1:** Inclusion and exclusion criteria for the study.

Inclusion Criteria	Exclusion Criteria
Diagnosis of Obsessive–Compulsive Disorder (according to DSM-5). Both sexes. Age ≥ 18 years and ≤65 years. Ability to provide valid written informed consent. For patients classified as responders to pharmacological treatment [R]: a clinical history of at least one pharmacological trial with an SRI for a minimum of 6 weeks and evidence of treatment response, defined as a 30% reduction in the Yale–Brown Obsessive Compulsive Scale (Y-BOCS) score relative to the patient’s baseline. For patients classified as resistant to pharmacological treatment [TR]: clear evidence of treatment resistance, defined as the absence of a significant clinical response after at least two treatment trials with selective serotonin reuptake inhibitors (SSRIs) and one trial with clomipramine, each administered for a minimum of 12 weeks at the maximum recommended dose.	Inability to provide informed consent. No clinical history of treatment with SRI medications. Clinical history of epilepsy or seizures. Presence of a pacemaker, removable metal prostheses, implanted medical pumps, or intracranial metal clips.

SRI: Serotonin reuptake inhibitors.

**Table 2:** Baseline sample characteristics and clinical outcomes.

Sample size	17
Sex (F:M)	6:11
Mean age (years)	35 ± 15
Dropouts	2
Y-BOCS (T0)	23.1 ± 6.8
Y-BOCS (T1)	20.6 ± 7.7
HAM-A (T0)	12.5 ± 7.6
HAM-A (T1)	10.9 ± 7.3
Partial responders	23.5% (*n* = 4)
Full responders	11.8% (*n* = 2)
Total responders	35.3% (*n* = 6)

Continuous variables are reported as mean ± standard deviation unless otherwise specified.

## Data Availability

The data presented in this study are available on reasonable request from the corresponding author. Although the data have been anonymized, they are not publicly available due to ethical, legal, and privacy restrictions, as they include sensitive genetic data and clinical information, in accordance with the informed consent provided by participants.
